# Confident but unsupported: auditing large language models against supplied evidence boundaries in drug-induced liver injury assessment

**DOI:** 10.3389/fpubh.2026.1904062

**Published:** 2026-08-05

**Authors:** Jinwan Shi, Yong Ma, Yinhui Liu, Bowen Shi

**Affiliations:** 1 Foshan Sanshui District People's Hospital, Foshan, Guangdong, China; 2 School of Investigation, Zhengzhou Police University, Zhengzhou, Henan, China; 3 School of Medical Information Engineering, Guangdong Pharmaceutical University, Guangzhou, Guangdong, China

**Keywords:** abstention, drug-induced liver injury, evidence grounding, large language models, pharmacovigilance, regulatory science, retrieval-augmented generation, selective prediction

## Abstract

**Introduction:**

Large language models (LLMs) are entering drug-safety and regulatory workflows, yet their behavior at the boundary between unavailable evidence and risk reassurance remains poorly characterized. Drug-induced liver injury (DILI) is a stringent setting because evidence is fragmented across labels, case reports, mechanistic studies, and curated knowledge bases, while unsupported low-risk reassurance can be consequential.

**Methods:**

We evaluated five LLMs on a fixed 47-drug DILI risk-assessment panel using 1,410 parsed responses from closed-book answering and an evidence-gated protocol that restricted responses to supplied PubMed-derived evidence. Curated DILI resources were excluded from prompts and used only for evaluation. After filtering, 38 drugs had no direct DILI decision-support evidence in the supplied packet, and 9 had direct DILI-relevant evidence; a post hoc PubMed title/abstract recall stress test identified five additional drugs with recoverable direct DILI evidence outside the packet.

**Results:**

In the no-direct-evidence slice defined by the supplied packet, closed-book models rarely abstained, with drug-level abstention ranging from 5.3% to 33.3%; the evidence-gated protocol required abstention, which all models followed for every no-direct-evidence drug. The same pattern held for recent or low-recognition drugs, where evidence-gated abstention reached 92.0% to 100.0% vs. 8.0% to 49.3% under closed-book answering. Closed-book models also produced high-confidence low-risk responses for DILI-positive drugs, a label-discordant pattern largely removed by evidence gating. Independent expert review of selected responses showed that label discordance did not always imply a clinically unreasonable low-risk category, but identified unsafe reassurance through overconfident wording and under-cautious responses in selected cases. When direct DILI evidence was provided, all models preserved citation-grounded non-abstaining answers. However, they differed in how often they committed to a conclusive rather than an uncertain risk category. Citation-bearing evidence-gated responses cited only the supplied PubMed identifiers and achieved 91.2% to 100.0% concordance with the supplied grade.

**Discussion:**

These findings identify unsupported reassurance as measurable evidence-boundary behavior in LLM drug-risk assessment and establish a reproducible framework for auditing adherence to an externally supplied evidence boundary, defined by PubMed evidence classification and enforced by prompt policy rather than inferred independently by the model.

## 1 Introduction

Large language models (LLMs) are increasingly being tested in medicine, drug development, and regulatory science because they can synthesize clinical text, answer biomedical questions, and support document-intensive workflows at a scale that is difficult for manual review alone (1–4). But this opportunity carries a central safety problem: clinically fluent answers can appear decisive even when the evidentiary basis is weak, unavailable, or outside the information provided to the user (5–8). For regulatory applications, this distinction is not cosmetic. Decisions about medicine safety depend on traceable evidence, uncertainty handling, and reproducible source attribution, rather than on the surface plausibility of a generated answer (4, 9, 10).

Pharmacovigilance is a natural yet demanding test bed for this problem. LLMs and related language models have been evaluated for literature screening, adverse-event extraction, drug–drug interaction assessment, social-media adverse-event detection, and regulatory document processing (11–16). Retrieval-augmented generation and prompt-based reasoning can improve access to external evidence and make model outputs more auditable (17–20). However, retrieval alone does not resolve whether a model should answer. A response can cite valid records while overstating indirect evidence, and a closed-book response can treat the absence of retrieved evidence as reassurance. These are evidence-boundary behaviors, not merely factual-error behaviors (8, 12, 21, 22).

Drug-induced liver injury (DILI) is a high-value domain for studying this boundary. DILI is a major cause of drug-development attrition, regulatory action, and post-marketing safety concerns. Yet its assessment is difficult because clinical phenotypes are heterogeneous, causality is often uncertain, and evidence is dispersed across labels, case reports, mechanistic studies, and curated knowledge bases (23–27). Several public resources summarize DILI risk or severity, including DILIrank 2.0, DILIst, LiverTox, and the Liver Toxicity Knowledge Base (28–32). These resources are valuable for evaluation, but they also pose a methodological hazard for LLM assessment. If such curated sources are visible to the model or embedded in the prompt, the task becomes a label-recall exercise rather than an evidence-based risk assessment.

Existing medical LLM evaluations have demonstrated strong clinical knowledge, examination performance, and potential utility in structured decision tasks (2, 5, 33, 34). More focused DILI work has evaluated LLM responses to hepatotoxicity questions, explainable LLM-assisted literature identification, and clinical-note pipelines for real-time DILI identification (35–37). These studies address clinically important but distinct tasks: question answering or triage, where the model is expected to use available knowledge; retrieval or literature identification; and extraction from patient records. They do not directly test whether a model should refrain from a drug-risk conclusion when the task-supplied evidence packet lacks direct DILI support. Simultaneously, recent studies emphasize persistent problems in calibration, hallucination, adversarial susceptibility, and insufficient expression of uncertainty (6–8, 21, 22). In drug safety, the critical unit of evaluation is therefore not only whether a model's answer is ultimately consistent with a hidden reference label. It is also a question of whether an externally supplied evidence packet licenses the answer under the rules of the task. This is especially important for new or low-recognition drugs, where sparse public evidence may reflect limited exposure, recency, or incomplete reporting rather than low biological risk.

We evaluate this evidence-boundary behavior in a fixed DILI risk-assessment task. We compared closed-book answers with an evidence-gated protocol across five LLMs, using the same drug panel, prompts, repeated queries, and response schema. In the primary analysis, DILI labels from curated resources were held out of model prompts and used only for evaluation. In the evidence-gated arm, each model received a PubMed-derived evidence packet containing classified direct DILI decision-support records, while curated proxy sources such as DILIrank, DILIst, LiverTox, and the Liver Toxicity Knowledge Base were excluded from the packet. This design makes four contributions. First, it operationalizes evidence-boundary behavior in DILI risk assessment under an externally supplied evidence boundary. Second, it separates prompt-supplied evidence from hidden reference labels, allowing label agreement and adherence to the supplied evidence boundary to be evaluated separately. Third, it quantifies abstention discipline alongside content-level endpoints, including citation containment, supplied-grade concordance, label-discordant low-risk reassurance, and negative-control over-warning, because abstention alone is not a complete safety assessment. Fourth, it provides a reproducible prompt, schema, response parsing, and source data package for auditing model behavior. The study therefore tests a practical regulatory-science requirement for medical LLMs: generated safety assessments should remain within the evidence supplied to the user.

## 2 Methods

### 2.1 Study design

**Figure 1:**
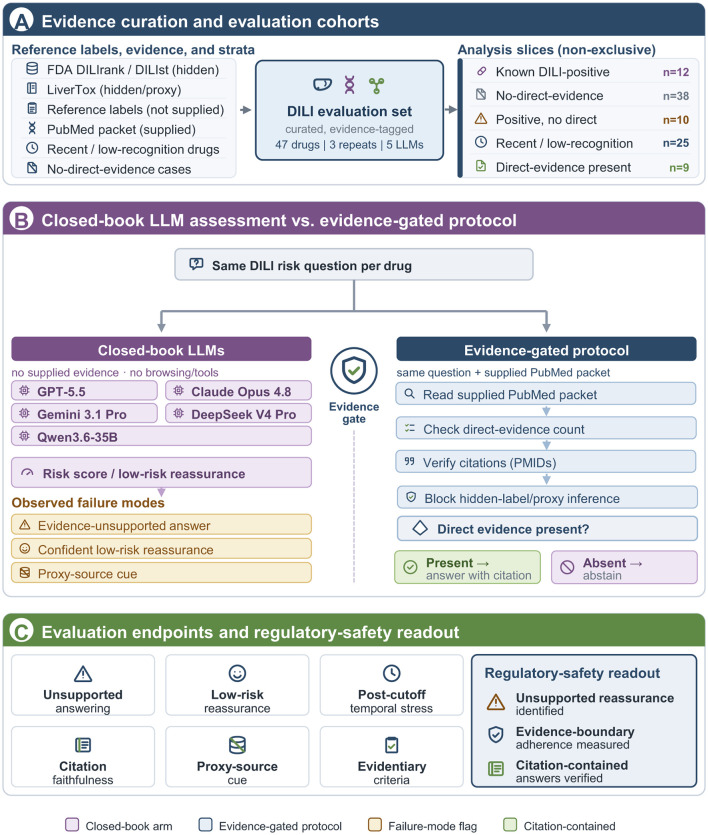
Study design. Panel **(A)** summarizes evidence curation, hidden reference labels, the supplied PubMed packet, and the overlapping evaluation cohorts. Panel **(B)** compares closed-book (CB) drug-induced liver injury (DILI) risk assessment with the evidence-gated (EG) protocol using the same fixed drug panel, prompts, repeats, and model roster. Panel **(C)** summarizes the evaluation endpoints and regulatory-safety readouts. LLM, large language model.

We conducted a prespecified comparative evaluation of large language model responses to questions about drug-induced liver injury (DILI) risk. The study compared two modes of use for each model: a closed-book mode, in which the model answered from its existing knowledge without external evidence, and an evidence-gated mode, in which the model could use only a supplied PubMed-derived evidence packet and was instructed to abstain when direct evidence was absent or insufficient (Figure 1). In the evidence-gated arm, the evidence boundary was externally defined by the PubMed evidence-classification pipeline and enforced by prompt policy; the experiment therefore audits adherence to a supplied evidence boundary rather than intrinsic model recognition of evidence absence. In the no-direct-evidence slice, the scientific contrast was protocol adherence in the evidence-gated arm vs. closed-book behavior under the same risk task; in the direct-evidence slice, it was whether the evidence-gated mode could cite and use supplied evidence when direct evidence existed. The unit of data generation was a model response; primary paired abstention analyses were conducted at the drug level after averaging the three repeats within each drug. Each drug was queried three times in each mode for each model, yielding 141 responses per mode and 1,410 total responses across five models.

The final evaluation used a fixed drug panel, fixed prompts, fixed response schema, fixed repeat structure, and fixed analysis rules. DILI reference datasets were used to define evaluation strata and hidden labels but labels from DILIrank, DILIst, LiverTox, or the Liver Toxicity Knowledge Base were not included in any model prompt.

### 2.2 Drug panel and evaluation strata

The evaluation panel comprised 47 drugs selected to stress clinically relevant evidence-boundary behavior rather than to estimate population-level DILI prevalence. The panel included 22 legacy calibration drugs with DILI-positive or DILI-negative status derived from FDA DILIrank 2.0 and DILIst, and 25 recent or low-recognition drugs intended to test behavior under limited public evidence and limited model familiarity. All quantitative rates should therefore be interpreted as conditional on this purposive stress-test panel rather than as prevalence estimates for all drugs or all LLM drug-safety outputs. The panel was fixed before model execution. Drug names were queried by generic or approved ingredient names when available; suffixes and product descriptors were retained in the panel metadata but were not used as hidden risk labels. DILIrank 2.0 is an FDA resource of 1,336 approved drugs categorized by DILI concern, based on FDA labeling and literature review (28, 29). DILIst is an FDA-linked binary DILI dataset of 1,279 drugs, including 768 positives and 511 negatives, derived by augmenting DILIrank with additional human hepatotoxicity datasets (30, 31). Legacy positive controls in this panel came from DILIst-positive provenance. Legacy negative or over-warning controls came either from concordant DILIrank-negative and DILIst-negative provenance, when both axes were available, or from DILIrank-negative provenance when no positive source label was used. We did not treat DILIrank less-concern or ambiguous tiers as deterministic negative labels in the submitted panel unless an explicit negative source axis was present. Recent drug candidates were derived from the public FDA CDER lists of approvals for new molecular entities and new therapeutic biologics (38). Hidden DILI labels for recent or low-recognition drugs were assigned based on LiverTox likelihood categories, with A/B treated as candidate-positive, D/E as candidate-negative, and C, other, or no-match categories as unresolved; these labels were used only for evaluation and were never supplied to prompts. Source-axis provenance and collapse counts are summarized in Supplementary Table S21.

The panel was analyzed using the following prespecified slices: all drugs; drugs with no direct DILI evidence in the supplied packet; DILI-positive drugs without direct evidence in the supplied packet; recent or low-recognition drugs; drugs with direct DILI evidence; and a small red-team slice containing direct adverse evidence or ambiguity requiring careful evidence handling. Because model knowledge cutoffs were disclosed inconsistently across providers, the recent or low-recognition slice was not treated as a universal model-cutoff-defined stratum; instead, it served as a prespecified stress test for limited public evidence and limited model familiarity. Full drug-level metadata, evidence-readiness categories, and slice membership are reported in Supplementary Table S1; panel-level composition is summarized in Supplementary Table S2, and model recency information is reported in Supplementary Table S4.

### 2.3 Evidence packet construction

In the evidence-gated mode, drug-specific evidence packets were constructed from PubMed title and abstract records. Retrieved records were filtered using drug-name matching and liver-injury relevance rules. Records were classified as direct DILI clinical or case evidence, direct nonclinical or mechanistic evidence, liver context not attributed to the queried drug, safety context only, protective or treatment liver context, safety-negative context, hepatitis background, low relevance, or forbidden proxy-source evidence. The phrase “no direct evidence” throughout the study refers to the absence of direct DILI decision-support evidence in the supplied PubMed title/abstract packet, not to a claim that no evidence exists anywhere in the literature, labels, or full-text sources. Records mentioning DILIrank, DILIst, LiverTox, or the Liver Toxicity Knowledge Base were excluded from the supplied evidence packet to prevent direct leakage from benchmark or label resources. LiverTox was also treated as a forbidden proxy-source name during output auditing because it is a curated DILI knowledge source rather than patient-level evidence supplied to the model (32).

For each drug, the evidence-gated prompt included the number of direct DILI decision-support records and the filtered evidence snippets. If the direct evidence count was zero, the prompt required abstaining or issuing an uncertain response and prohibited treating the absence of evidence as a low-risk safety claim. If direct evidence was present, the model could provide a non-abstaining risk assessment but had to cite only PubMed identifiers in the supplied packet. Thus, the license to answer in the evidence-gated arm came from the upstream evidence-classification pipeline and the prompt rule, not from an autonomous model decision that no evidence existed. Evidence filtering classes are reported in Supplementary Table S3, and drug-level supplied and direct PubMed identifiers are summarized in Supplementary Table S6.

To characterize evidence-channel recall, we added a PubMed title/abstract recall stress test to quantify whether direct DILI decision-support records were recoverable from PubMed but absent from the supplied packet. This audit repeated title/abstract queries for all 47 drugs, applied high-specificity title/abstract filters for drug-bound DILI evidence, and manually adjudicated candidate missed records. The audit did not use full-text retrieval, product labels, spontaneous-report data, or curated DILI resources. It therefore tested only the recall of the title/abstract evidence packet used as a prompt. We summarized precision and recall for this restricted channel: broad supplied-record precision was assessed on sampled supplied direct records, whereas drug-level packet recall was calculated against the augmented set of submitted-packet direct-evidence drugs plus newly identified title/abstract direct-positive drugs. Drugs newly identified as having direct title/abstract evidence were carried into sensitivity slice definitions but were not used to change the original model prompts or responses (Supplementary Tables S13, S22).

### 2.4 Model panel and prompting

Five large language models were evaluated: GPT-5.5, Claude Opus 4.8, Gemini 3.1 Pro Preview, DeepSeek V4 Pro, and Qwen3.6-35B-A3B. For compact display, Gemini 3.1 Pro Preview is abbreviated as Gemini 3.1 Pro and Qwen3.6-35B-A3B as Qwen3.6-35B in figures and compact tables. The same 47-drug panel, two-arm design, prompt order, and three-repeat structure were used for all models. Closed-book prompts provided only the drug name and an anchored 0–100 DILI risk-score scale. They instructed the model not to browse, use tools, cite nonexistent evidence, infer risk from drug familiarity, or use hidden label databases. Evidence-gated prompts supplied the filtered PubMed evidence packet and required strict use of the supplied evidence only.

All prompts required a strict JSON response containing the drug name, 0–100 risk score, risk category, abstention flag, abstention reason, knowledge or evidence basis, PubMed identifiers where applicable, evidence summary, counterevidence summary, and confidence score. Model provider, model identifier, disclosed knowledge cutoff status, decoding settings, output length limits, retry handling, response-file provenance, and prompt hashes are reported in Supplementary Table S4 and the release metadata; response-file provenance is visualized in Supplementary Figure S4. OpenAI and Anthropic calls used provider-default decoding values returned or accepted by their official interfaces, whereas Gemini, DeepSeek, and Qwen were run at a temperature of 0.2. The prompts, panel, repeats, schema, and analysis rules were otherwise held constant across models. Prompt templates and the response schema are provided in Supplementary Methods.

The primary five-model responses were generated on 5 June 2026 UTC. The risk-score scale was explicitly anchored in each prompt: 0 indicated no credible clinical evidence of clinically meaningful DILI; 25 indicated rare or weakly suspected DILI; 50 indicated plausible but uncertain or mixed evidence; 75 indicated known clinically meaningful DILI with credible reports, warnings, or mechanistic plausibility; and 100 indicated well-established frequent or severe DILI liability, severe hepatotoxicity, liver-failure risk, or a strong regulatory warning. The score was treated as an ordinal model-reported field, rather than a calibrated probability.

After the primary five-model analysis, we conducted a prompt-ablation sensitivity experiment using DeepSeek V4 Pro and Qwen3.6-35B-A3B to isolate four potential drivers of the evidence-gated effect: stronger closed-book uncertainty wording, retrieval-only evidence packets without upstream evidence-class labels, evidence-gated instructions without an explicit direct-evidence count, and the full evidence-gated prompt. These ablations used the same 47 drugs, three-repeat structure, JSON schema, and response parser as the primary analysis, but were treated as sensitivity analyses rather than as part of the primary five-model comparison (Supplementary Table S7).

As a post hoc sensitivity analysis, we added a curated reference-label evidence block to the original evidence-gated packet for the 32 non-unresolved drugs and reran the two low-cost models. This analysis was not part of the primary five-model comparison. It was used only to test whether abstention in the primary evidence-gated arm reflected the protocol-defined absence of supplied direct evidence rather than an inability to use explicit reference-label evidence. Coarse label responsiveness and citation containment were summarized in Supplementary Table S12.

We also ran two limited low-cost API sensitivity pilots in DeepSeek V4 Pro and Qwen3.6-35B-A3B after the primary analysis. A matched-decoding pilot compared temperature 0.0 with temperature 0.2 under identical model, drug, arm, and prompt conditions; a drug-name-form pilot compared generic-name prompts with brand or synonym-based prompts in closed-book mode. These pilots were deliberately restricted to the two low-cost models because the provider interfaces did not expose identical decoding controls across all five evaluated models, and because the pilots were intended to test whether the main qualitative behavior was obviously brittle rather than to create a second full five-model experiment (Supplementary Table S16).

### 2.5 Outcomes

The primary endpoint was abstention discipline in the no-direct-evidence slice. For each model, we compared the proportion of closed-book responses that abstained with the proportion of evidence-gated responses that abstained when no direct DILI evidence was provided. Because the evidence-gated prompt required abstention when the direct evidence count was zero, the evidence-gated value in this slice is interpreted as protocol adherence rather than spontaneous model self-restraint. In this slice, a non-abstaining answer was considered unsupported if it made a substantive DILI risk judgment or safety reassurance despite the absence of directly supplied evidence; explicit abstention or explicit uncertainty was not counted as unsupported.

Secondary endpoints assessed evidence faithfulness and safety-relevant response behavior. Citation containment was defined at the response level as the proportion of citation-bearing evidence-gated responses for which all cited PubMed identifiers were present in the supplied evidence packet. Supplied-grade concordance was defined as the consistency between the model's stated evidence grade and the class of the cited evidence. Because evidence classes were provided in the prompt, this endpoint measures adherence to the supplied evidence labels rather than autonomous assessment of evidence strength. Both citation containment and supplied-grade concordance were computed among citation-bearing evidence-gated responses across the full panel; these denominators are response counts, not counts of direct-evidence drugs or PMID mentions. Direct-evidence conclusive-category answer retention was defined as the response-level proportion of evidence-gated repeated queries in the direct-evidence slice that were non-abstaining and assigned a definite (low, moderate, or high) risk category rather than an uncertain category; consistent with the primary analysis, which treats explicit uncertainty as evidence-appropriate non-commitment, this endpoint is intentionally stricter than non-abstention, so a response that did not abstain but selected an uncertain risk category was counted as non-abstaining but not as conclusive. Formal non-abstention in the direct-evidence slice was 100.0% across all five models, so differences in conclusive-category retention reflect how often a model committed to a definite risk category rather than abstained. Proxy-source leakage was defined as an output mention of LiverTox, DILIrank, DILIst, or the Liver Toxicity Knowledge Base. Label-discordant low-risk reassurance was defined in labeled slices as a non-abstaining low-risk response for a DILI-positive drug. High-confidence low-risk reassurance additionally required model-reported confidence_0_100 ≥70 and a risk score ≤ 25; because both thresholds use model-self-reported fields, the resulting counts are not externally calibrated probability estimates (6). High-risk responses were defined as those for which the model-reported risk_category field was equal to high. Negative-control over-warning was defined as a high-risk response for a legacy DILI-negative or over-warning control. A tier-aware red-team endpoint assessed whether the model acknowledged direct adverse evidence while respecting ambiguity for nonclinical, review-tier, co-suspect, or counterevidence-containing records. This red-team endpoint contained nine responses per model and was treated as an exploratory stress test rather than as a model-ranking endpoint. Outcome definitions are summarized in Supplementary Table S8.

### 2.6 Response parsing and quality control

Responses were parsed as JSON using the fixed schema. Responses with valid JSON embedded in surrounding text were recovered when all required fields could be extracted without altering the model's substantive values. Rows requiring provider retry due to malformed or truncated output were tracked separately from initially valid rows. The final analysis retained only one response per model, drug, arm, and repeat after merging attempts. Prompt files were scanned for Chinese characters and forbidden proxy-source names before execution. Outputs were scanned for forbidden proxy-source terms, missing or off-packet PubMed identifiers, and response-schema violations.

### 2.7 Independent expert review of selected responses

To test whether the operational endpoints aligned with clinically meaningful judgments, two expert reviewers independently assessed a purposively selected set of responses using the same scoring sheet. The reviewers were not involved in model prompting, response parsing, or primary endpoint calculation. The packet included 24 closed-book, low-risk responses; 20 responses in the direct-evidence slice; 15 evidence-gated abstentions; and six high-risk or over-warning responses. Cases were selected before reviewer scoring to cover the principal operational endpoint categories, multiple models, and both positive and negative or control strata; they were not selected based on reviewer judgments. This review was designed as a clinical calibration of endpoint interpretation rather than a prevalence-estimating sample. The review packet did not explicitly label the model identity, hidden reference label, or response arm, although the response arm could, in some cases, be inferred from abstention behavior or citation content; reviewers were instructed not to search the web while scoring. Reviewers assessed whether the risk category was clinically reasonable, whether a low-risk response provided inappropriate reassurance, whether a high-risk response provided inappropriate over-warning, whether the model should have abstained, and which risk category they preferred. The review was intended to separate categorical risk reasonableness from communication-safety concerns; it was not used to change model outputs or reference labels. Inter-reviewer agreement was summarized with percent agreement, Cohen's κ, and Gwet's AC1 because several axes were highly imbalanced and κ can be unstable in such settings. Expert-level counts are reported in Supplementary Table S11; agreement statistics are reported in Supplementary Table S15; case-level reasons are provided in the machine-readable source data.

### 2.8 Statistical analysis

All primary analyses were conducted at the drug level to avoid treating repeated prompts for the same drug as independent observations. For each model and slice, repeat-level outcomes were first averaged within each drug. We then estimated the mean abstention rate in each arm and the paired difference between evidence-gated and closed-book modes. Uncertainty intervals for paired differences were computed using a drug-clustered nonparametric bootstrap. These paired analyses quantify adherence to a prespecified evidence-boundary protocol and contrast it with closed-book behavior. The five model-specific intervals are reported as descriptive evidence of consistency across the evaluated model roster; no single model was designated as a primary model, and no omnibus or multiplicity-adjusted confirmatory test was used. Response-level proportions are reported descriptively in the main text. When uncertainty intervals are shown for response-level endpoints, they use a drug-clustered bootstrap rather than row-wise binomial intervals, so that repeated responses for the same drug are not treated as independent (Supplementary Table S18).

Risk-score analyses treated the model-reported 0–100 score as an ordinal risk score rather than as a calibrated probability (6). We therefore used scores only for predefined threshold-based summaries, including high-confidence label-discordant low-risk reassurance and negative-control high-risk over-warning, without interpreting them as clinical probabilities. We also evaluated a threshold grid for the high-confidence low-risk endpoint using confidence cutoffs of 60, 70, 80, and 90 and risk-score cutoffs of 20, 25, and 30 (Supplementary Table S17). Sensitivity analyses assessed repeat-level agreement (Supplementary Figure S2), model-specific response provenance, retry effects for Gemini (Supplementary Figure S1), forbidden-proxy outputs, label-inclusive evidence gating in two low-cost models, matched-decoding pilots, brand/synonym prompts, and an exploratory utility analysis of an expert subset. The utility analysis scored correct substantive answers, appropriate abstentions, harmful low-risk reassurance, harmful over-warning, and unnecessary abstention or uncertainty under balanced, harm-averse, and answer-preferring weights; it is reported as a sensitivity analysis rather than as a clinical decision-curve analysis (Supplementary Table S19). Reporting was audited against a TRIPOD-LLM-oriented checklist (39), which is provided in the source data and summarized in Supplementary Table S20. Repeat-level agreement was reported to indicate response stability, but inferential analyses continued to use drugs rather than repeats as the units of analysis.

## 3 Results

### 3.1 A fixed DILI panel separated evidence absence from evidence presence

We evaluated 47 drugs across two response modes and five models, yielding 1,410 parsed responses. The panel was designed to separate two questions that are often conflated in medical LLM evaluation. Whether a model can discuss a known safety topic, and whether it respects the boundary of the evidence actually supplied to it. After PubMed evidence filtering, 38 drugs had no direct DILI decision-support evidence in the supplied title/abstract packet, while 9 drugs had at least one direct DILI-relevant record. This structure created a primary no-direct-evidence slice for testing unsupported answering, and a smaller direct-evidence slice for checking whether the evidence-gated protocol merely refused or could also act on the supplied evidence. All rates below are conditional on this purposively selected stress-test panel.

The recall stress test showed that the evidence packet was specific but not exhaustive. Across 1,804 PubMed title/abstract hit rows, 78 candidate missed records were manually adjudicated; 18 candidate rows, corresponding to five additional drugs, were counted as direct-positive at the title/abstract level (Supplementary Table S13). Relative to the augmented title/abstract direct-positive set, the submitted packet captured nine of 14 direct-positive drugs (64.3%). At the record level, the supplied packet contained 13 direct records, while the recall audit found 18 additional adjudicated direct-positive title/abstract rows, yielding a restricted-channel capture of 13 of 31 direct-positive rows (41.9%). Precision of the supplied direct-record sample was 13 of 13 under a broad direct-evidence definition and 10 of 13 (76.9%) under a stricter clinical or pharmacoepidemiology definition; the high-specificity missed-candidate screen yielded 18 direct-positive rows among 78 adjudicated candidates (23.1%; Supplementary Table S22). This changed the interpretation of the no-direct-evidence slice from the absence of global evidence to the absence of direct evidence in the supplied packet. Four of the five newly identified drugs were legacy DILI-positive controls, reducing the legacy DILI-positive/no-direct subset from 8 to 4 after the recall audit; the fifth was primaquine, a legacy negative/over-warning control for which the audit found recoverable title/abstract DILI evidence. The original prompts and model responses were not changed, so the primary analysis remains an audit of behavior under the evidence actually supplied to the models.

### 3.2 Closed-book models were usually answered when direct DILI evidence was absent

**Figure 2:**
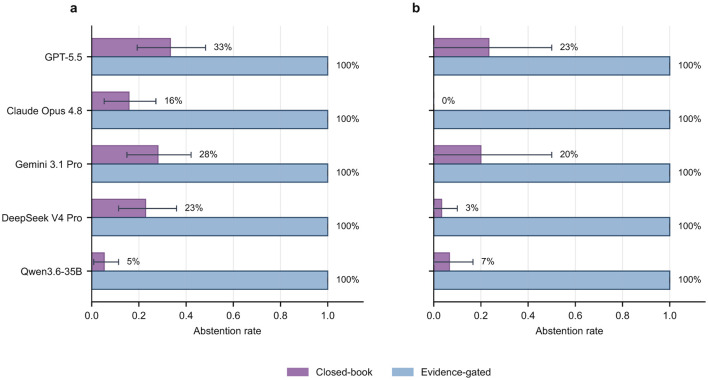
Abstention outcomes in no-direct-evidence slices defined by the supplied evidence packet. Panel **(a)** shows drug-level mean abstention for 38 drugs without direct DILI decision-support evidence in the supplied packet. Panel **(b)** shows the same endpoint for the original 10 positive or candidate-positive drugs without direct supplied evidence, comprising eight legacy DILI-positive controls and two recent candidate-positive drugs; a *post hoc* PubMed title/abstract recall audit reclassified four of the legacy-positive drugs as having recoverable direct title/abstract evidence outside the supplied packet. These drugs and their slice labels are listed in Supplementary Table S1. Error bars show drug-clustered bootstrap 95% intervals for the mean abstention rate.

In the no-direct-evidence slice defined by the supplied packet, closed-book models rarely abstained. At the drug level, mean closed-book abstention ranged from 5.3% to 33.3% across the five models (Table 1; Figure 2; Supplementary Table S5). In the evidence-gated mode, the protocol required abstention when no direct evidence was supplied, and all five models followed this instruction for every no-direct-evidence drug. The paired drug-level difference between evidence-gated and closed-book abstention ranged from 66.7 to 94.7 percentage points; the model-specific bootstrap intervals are reported descriptively, and all excluded zero (Supplementary Figure S3). The same pattern was observed in the original positive/no-direct-evidence composite slice defined by the supplied packet, which contained eight legacy DILI-positive controls and two recent candidate-positive drugs. After the PubMed title/abstract recall audit, four legacy DILI-positive drugs still lacked recoverable direct title/abstract evidence; in this stricter post-recall subset, evidence-gated abstention remained 100.0% for every model, while closed-book abstention was 0.0% for Claude Opus 4.8, Gemini 3.1 Pro, DeepSeek V4 Pro, and Qwen3.6-35B, and 8.3% for GPT-5.5. When the two recent candidate-positive/no-direct drugs were added to form the post-recall 6-drug positive/no-direct sensitivity slice, evidence-gated abstention again remained 100.0%, while closed-book abstention ranged from 0.0% to 38.9% across models. This indicates that the finding was not driven solely by obviously low-risk or unfamiliar drugs and shows that the denominator depends on the evidence-retrieval channel. Because abstention in this arm was specified by the evidence-gated policy, this contrast should be interpreted as protocol adherence vs. closed-book evidence-boundary behavior, not as calibration of spontaneous abstention.

**Table 1:** Primary evidence-boundary outcomes by model.

Model	Closed-book abstention	Evidence-gated abstention	Difference, pp (95% CI)	Low-risk reassurance (CB → EG)	Conclusive-category answer retention	Supplied-grade concordance
GPT-5.5	33.3%	100.0%	66.7 (51.8–80.7)	6 → 0	88.9%	100.0% (110)
Claude Opus 4.8	15.8%	100.0%	84.2 (72.8–94.7)	8 → 0	100.0%	100.0% (98)
Gemini 3.1 Pro	28.1%	100.0%	71.9 (57.9–85.1)	12 → 0	96.3%	91.2% (34)
DeepSeek V4 Pro	22.8%	100.0%	77.2 (64.0–88.6)	12 → 0	96.3%	97.4% (115)
Qwen3.6-35B	5.3%	100.0%	94.7 (88.6–99.1)	12 → 1	25.9%	99.2% (120)

Abstention rates are drug-level means in the 38-drug no-direct-evidence slice. Differences compare evidence-gated vs. closed-book abstention with drug-clustered bootstrap 95% intervals. Low-risk reassurance counts are high-confidence low-risk responses among 12 legacy DILI-positive drugs and are interpreted as label-discordant, not as expert-adjudicated clinical falsehoods. Conclusive-category answer retention is the response-level proportion of evidence-gated repeated queries in the 9-drug direct-evidence slice that were non-abstaining and assigned a definite (low, moderate, or high) risk category rather than an uncertain category. No model formally abstained on any direct-evidence query (formal non-abstention 100.0% for all five models); the lower value for Qwen3.6-35B therefore reflects frequent uncertain categories, not abstention. These model-specific values are descriptive process checks and should not be interpreted as a statistically supported model ranking because no omnibus or multiplicity-adjusted model comparison was performed. Supplied-grade concordance is computed against supplied evidence-class labels among citation-bearing evidence-gated responses across the full panel; parenthetical values are citation-bearing response counts. Qwen3.6-35B denotes Qwen3.6-35B-A3B.

### 3.3 Closed-book answers included label-discordant low-risk reassurance

**Figure 3:**
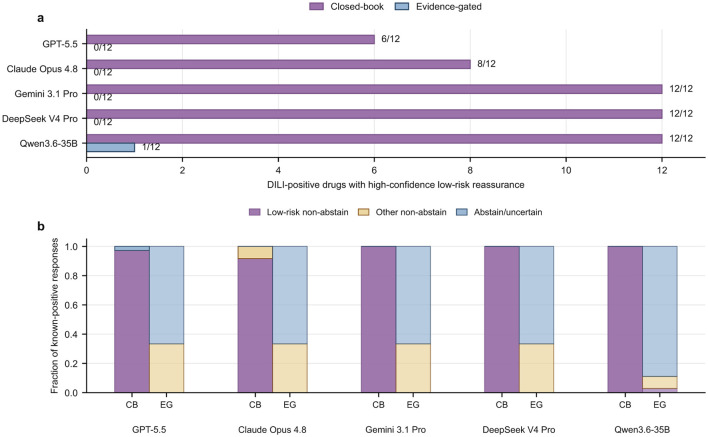
Label-discordant low-risk reassurance in the legacy DILI-positive slice. Panel **(a)** shows the number of DILI-positive drugs, of 12, with at least one high-confidence low-risk response, defined as a non-abstaining low-risk answer with model-reported confidence_0_100 ≥70 and risk score ≤ 25. The 12 legacy DILI-positive drugs were aliskiren, amisulpride, betahistine, bupivacaine, celiprolol, cetirizine, chlorothiazide, clobetasol, fondaparinux, losartan, metformin, and octreotide. Panel **(b)** shows the response disposition for the 36 repeat responses per model and mode in this 12-drug slice. CB, closed-book; EG, evidence-gated.

A central evidence-boundary pattern was confident low-risk reassurance for drugs that were positive in the hidden DILI reference labels. Across labeled slices, closed-book models produced high-confidence low-risk responses for DILI-positive drugs in every model, whereas the evidence-gated mode eliminated this pattern in four models and left one such response in the fifth model (Figure 3). This was most pronounced in the legacy DILI-positive calibration slice, where several closed-book models repeatedly assigned low risk despite the hidden positive label. The result is therefore best interpreted as reassurance unsupported by the task-supplied evidence and discordant with the reference label, rather than as a calibrated estimate of true DILI probability; the expert review below further separated broad risk-category reasonableness from overconfident risk communication.

### 3.4 Expert review separated risk-category judgment from reassurance quality

The expert review supported a more granular interpretation of the low-risk reassurance endpoint. Among 24 sampled closed-book, low-risk responses, Expert reviewer 1 judged all risk categories to be clinically reasonable and flagged no inappropriate low-risk reassurance. Expert reviewer 2 applied a stricter standard, judging 22 of 24 risk categories clinically reasonable but flagging 3 of 24 responses as inappropriate low-risk reassurance (Supplementary Table S11). These three flags were heterogeneous. One aliskiren response was judged to be in a defensible low-risk category but to provide excessive reassurance because it paired a zero score and high confidence with liver-safety wording. Two bupivacaine responses were judged as category-level disagreements: Expert reviewer 2 preferred a moderate rating and judged that the model should have abstained, citing rare but reported cholestatic DILI. Agreement was high across several axes but not uniform: in the closed-book, low-risk subset, percent agreement was 91.7% for clinical reasonableness and 83.3% for inappropriate low-risk reassurance; in the direct-evidence subset, agreement was 85.0% for whether the answer was supported by the supplied evidence and 80.0% for risk-category reasonableness (Supplementary Table S15).

Thus, within this purposive review set, DILI-positive reference labeling did not, by itself, render every sampled low-risk category clinically incorrect. Instead, the review identified two ways in which closed-book, low-risk responses can become unsafe: overconfident wording around an otherwise defensible low-risk classification, and assigning a low-risk category when rare clinical DILI reports warrant greater uncertainty. The 2 of 24 should-abstain judgments were therefore answer-entitlement judgments as well as category disagreements; they concerned whether the model had sufficient direct evidence to provide a substantive risk assessment. Because several expert-review axes were small and imbalanced, the agreement statistics in Supplementary Table S15 are best interpreted as calibration of endpoint interpretation rather than as population estimates of clinical error. This review was not designed to estimate the prevalence of clinically inappropriate reassurance across the full set of responses.

The same review supported the evidence-gated abstention logic. Both reviewers judged all 15 sampled evidence-gated abstentions as cases in which the model should have abstained. Agreement was lowest in the small high-risk or over-warning subset: one reviewer judged 1 of 6 responses as inappropriate over-warning, whereas the other judged all 6 as inappropriate over-warning. These exploratory cases reinforce the need to evaluate evidence entitlement, risk communication, and categorical risk labels separately.

### 3.5 Negative-control drugs exposed the complementary over-warning pattern

The panel also included 10 legacy DILI-negative or over-warning controls. In closed-book mode, all models answered substantively for these controls, and two models produced high-risk responses: Qwen3.6-35B produced 3 high-risk responses involving 1 of 10 drugs, and DeepSeek V4 Pro produced 4 high-risk responses involving 2 of 10 drugs. The other three models produced no high-risk responses in this slice. In the evidence-gated mode, all five models produced no high-risk responses for the same 10 controls. Across models, 21 of 30 responses per model abstained, and the remaining responses stayed below the high-risk threshold (Supplementary Table S10). A sensitivity calculation across the broader 18-drug negative or candidate-negative control set showed the same direction: high-risk responses occurred in the closed-book mode but not in the evidence-gated mode. Thus, within these control slices, the protocol directionally reduced both evidence-boundary patterns evaluated in this study: low-risk reassurance for hidden-positive drugs and unsupported high-risk over-warning for negative or control drugs.

### 3.6 Recent or low-recognition drug stress testing showed the same evidence-boundary behavior

**Figure 4:**
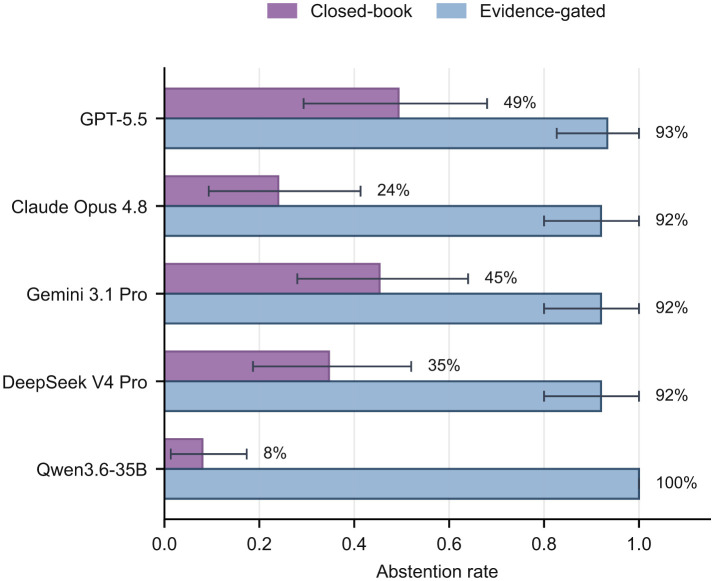
Recent/low-recognition stress-test slice. Drug-level mean abstention is shown for 25 drugs selected to limit reliance on model familiarity. Error bars show drug-clustered bootstrap 95% intervals.

Recent and low-recognition drugs served as a stress test of reliance on model familiarity, not a claim that every drug fell after every model's disclosed knowledge cutoff. In this slice, closed-book models continued to issue substantive DILI risk judgments for many drugs, with drug-level abstention ranging from 8.0% to 49.3%. The evidence-gated mode again largely abstained when direct DILI evidence was absent, with drug-level abstention of 92.0% to 100.0% across models (Figure 4). Conversely, it was not confined to older, familiar drugs present in curated DILI resources.

### 3.7 The evidence-gated protocol preserved evidence use when direct evidence was supplied

**Figure 5:**
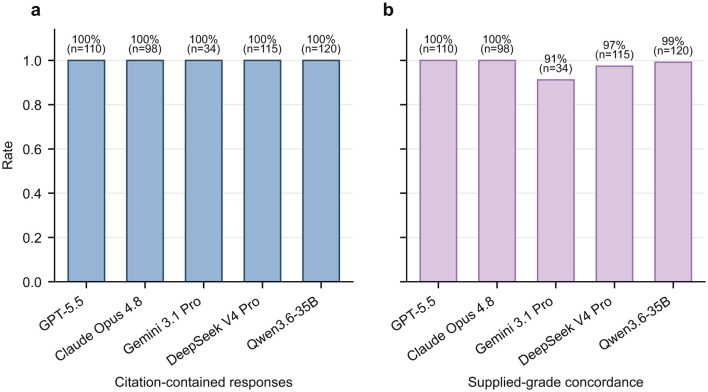
Evidence-gated citation containment and supplied-grade concordance. Panel **(a)** shows the proportion of citation-bearing evidence-gated responses for which all cited PubMed identifiers were present in the supplied packet. Panel **(b)** shows the proportion of citation-bearing evidence-gated responses whose stated evidence grade was concordant with the supplied evidence class across the full panel. Denominators above bars indicate citation-bearing response counts, not the number of direct-evidence drugs.

The direct-evidence slice tested whether evidence gating preserved substantive answering when direct DILI evidence was supplied. When direct DILI evidence was supplied, no model formally abstained on any direct-evidence query (formal non-abstention 100.0% across models). However, models differed in how often they committed to a definite risk category rather than an uncertain one after engaging with the supplied evidence (Table 1; Supplementary Table S18). This endpoint is therefore best read as a response-style and evidence-commitment measure, not as a model ranking. Among citation-bearing evidence-gated responses, citation containment was complete at the response level: all cited PubMed identifiers were contained in the evidence packet supplied to that response (Figure 5). Supplied-grade concordance, which measures adherence to the supplied evidence class rather than autonomous evidence grading, ranged from 91.2% to 100.0% among citation-bearing evidence-gated responses across the full panel. Citation-bearing rates differed by model, from 24.1% in Gemini 3.1 Pro to 85.1% in Qwen3.6-35B (Supplementary Table S14). These are response-level denominators and should not be read as the number of direct-evidence drugs. The non-zero evidence-gated high-risk responses in GPT-5.5 (six responses) and Gemini 3.1 Pro (five responses) occurred only for legacy DILI-positive drugs with direct clinical evidence in the packet: bupivacaine, cetirizine, and losartan for GPT-5.5, and cetirizine and losartan for Gemini 3.1 Pro. They therefore represent high-risk calls in direct-evidence positive cases rather than negative-control over-warning or no-direct-evidence protocol violations. These results indicate that the protocol improved abstention discipline in the absence of evidence while retaining the ability to cite supplied evidence when direct DILI-relevant records were present.

### 3.8 Sensitivity analyses supported the protocol interpretation

In the two-model prompt-ablation sensitivity analysis, stronger wording of closed-book uncertainty increased abstention but did not eliminate label-discordant low-risk responses in the positive/no-direct-evidence slice. Retrieval-only prompts that provided PubMed snippets but omitted upstream evidence-class labels produced substantially higher abstention in the no-direct-evidence slice, and the evidence-gated prompt without an explicit direct-evidence count remained close to the full evidence-gated protocol (Supplementary Table S7). These results support the interpretation that the main effect was driven by externally supplied evidence-boundary constraints applied to the prompt, rather than by model identity alone or by an explicit direct-count field alone. The high-confidence low-risk pattern was also stable across a grid of confidence and risk-score thresholds: across legacy-positive slices, no threshold setting produced a reversal in which the evidence-gated arm exceeded the closed-book arm in the number of evaluated models (Supplementary Table S17).

As a post hoc, label-inclusive sensitivity analysis, we added curated reference-label evidence to the original evidence-gated packet only for the two low-cost models. In this label-inclusive arm, both models stopped abstaining across all 32 non-unresolved tested drugs while maintaining complete citation containment. Coarse label responsiveness, defined using a permissive score threshold rather than clinical category adjudication, was 92.9%–100.0% in positive or candidate-positive strata and 85.2%–100.0% in negative or candidate-negative strata (Supplementary Table S12). A stricter positive-category version, which additionally required a moderate or high category, was 100.0% for DeepSeek V4 Pro and 26.2% for Qwen3.6-35B. This sensitivity supports the interpretation that the primary evidence-gated abstention reflected the protocol-defined absence of supplied direct evidence, not a fixed inability to respond when explicit reference-label evidence was supplied; it should not be interpreted as clinical risk-category validation.

The low-cost API sensitivity pilots did not indicate that the qualitative findings depended on a single temperature setting or a single drug-name form. In matched temperature 0.0 vs. 0.2 pairs, category agreement was 93.8% for DeepSeek V4 Pro and 81.2% for Qwen3.6-35B-A3B, with abstention agreement of 93.8% and 100.0%, respectively. In generic-name vs. brand/synonym pairs, both models showed 87.5% category agreement and 100.0% abstention agreement (Supplementary Table S16). These pilots are not a substitute for a full five-model rerun with identical decoding controls, but they provide a bounded robustness check for the main protocol.

### 3.9 Residual errors were concentrated in evidence grading and proxy-source leakage

The evidence-gated mode removed mentions of forbidden proxy sources used for hidden labeling or DILI knowledge curation. Conversely, two closed-book responses cited a forbidden proxy source despite being instructed not to cite external evidence (Supplementary Table S9). The remaining evidence-gated errors were not primarily off-packet citations but rather over- or under-grading the strength of the supplied evidence in a minority of responses. The exploratory red-team slice contained only nine responses per model, so we did not use it for model-specific quantitative comparison or as a model-ranking endpoint. Qualitatively, the residual failures involved handling ambiguous, indirect, nonclinical, co-suspect, or counterevidence-containing safety records, indicating that evidence grounding reduced but did not eliminate the need for structured evaluation of how models handle indirect safety evidence.

## 4 Discussion

This study identifies a specific evidence-boundary behavior in large language models for drug-safety assessment. When direct evidence was absent from the supplied record, closed-book models often converted uncertainty into a substantive judgment of DILI risk. A central form of this behavior was low-risk reassurance for drugs that were positive in hidden DILI reference labels. The finding does not imply that LLMs are uniformly unable to discuss hepatotoxicity, nor that the models independently inferred the evidence boundary in the evidence-gated arm. Rather, it shows that fluent closed-book answers can exceed the evidentiary basis available to the user, and that an externally defined evidence boundary can make adherence to that basis measurable.

The result fits a broader pattern in medical LLM evaluation. Prior research has shown that LLMs can encode substantial clinical knowledge (2). Yet they still fail in realistic clinical decision-making settings that require adherence to guidelines, information gathering, numerical interpretation, or reliable handling of uncertainty (5). Reviews of LLMs in medicine have therefore emphasized that impressive language and examination performance do not eliminate the need for task-specific safety evaluation (3). Our contribution is narrower and more regulatory: we isolate an evidence-boundary behavior in a pharmacovigilance setting, where the distinction between “no supplied evidence” and “evidence of no risk” is central to safety interpretation.

For regulatory science, this separation has direct consequences. Drug-induced liver injury evidence is fragmented across product labels, case reports, mechanistic studies, curated knowledge bases, and post-marketing surveillance. In such a setting, a model that gives a confident, low-risk answer without direct supporting evidence may appear helpful while weakening the evidentiary discipline on which pharmacovigilance depends. This is especially relevant for newer products and low-recognition drugs, where the absence of accessible evidence may reflect recency, limited exposure, or incomplete reporting rather than genuine safety concerns. The evidence-gated protocol tested here addresses this behavior procedurally: an external PubMed title/abstract classification pipeline defines whether direct evidence is present, and the prompt policy requires the model to answer only within that packet and to abstain when the packet lacks direct DILI evidence.

The independent expert review sharpened this interpretation and revealed variation in clinical tolerance for low-risk language. One reviewer judged all sampled low-risk categories clinically reasonable and flagged no inappropriate reassurance. A stricter reviewer flagged three low-risk responses: one as over-absolute reassurance despite an acceptable low-risk category, and two bupivacaine responses as under-cautious categories given rare but reported cholestatic DILI. This distinction matters for regulatory use. A broad low-risk classification may be defensible, yet the surrounding language can still create a stronger sense of safety than the evidence warrants; in other cases, rare clinical evidence may warrant an uncertain or moderate answer rather than a low-risk one.

The protocol is procedural rather than predictive. Its value is that it turns an implicit evidence problem into an explicit output requirement and makes adherence to that requirement measurable. In this design, evidence-gated abstention is a protocol-adherence outcome rather than a claim of spontaneous model self-restraint or autonomous evidence-boundary recognition. Abstention discipline is therefore necessary for the safety claim, but not sufficient on its own: the content of non-abstaining answers must also be assessed. When direct evidence was supplied, most models remained capable of providing citation-grounded, non-abstaining answers with complete citation containment and high supplied-grade concordance. However, one model more often returned an uncertain risk category than a conclusive one. A useful safety interface should not maximize abstention. It should reserve judgment when evidence is absent, cite faithfully when evidence is present, and preserve uncertainty when evidence is indirect or ambiguous. The protocol therefore requires two coupled audits: adherence to the externally supplied evidence boundary in the absence of evidence, and, when direct evidence is supplied, both non-abstention and the model's willingness to commit to a conclusive risk category.

The findings also have implications for how medical LLMs should be evaluated. General medical benchmarks, licensing-style questions, or clinical preference ratings may miss evidence-boundary behavior because they typically score answer plausibility or factual correctness rather than whether a model was entitled to make the claim given the evidence available in the task. Pharmacovigilance adds another layer: benchmark labels and curated DILI resources may themselves be sources of leakage. Evaluation protocols should therefore distinguish hidden labels from supplied evidence, audit references against the supplied record, and report abstention behavior as a first-class safety outcome. These requirements align with calls for more reproducible and transparent LLM evaluation in healthcare research (9) and with the need for domain-specific safety metrics in pharmacovigilance applications (11).

Several design features define the scope of the present study. The drug panel was intentionally stress-test-oriented rather than prevalence-representative: it was built to expose reassurance unsupported by the task-supplied evidence and to identify temporal evidence gaps, not to estimate the population frequency of unsafe LLM responses across all drugs. The expert review was likewise a purposive calibration exercise rather than a prevalence-estimating adjudication study. It involved two reviewers, showed differences in tolerance for low- and high-risk language, and should therefore be interpreted as supporting the interpretation of endpoints rather than as a replacement for formal DILI causality adjudication. DILI-positive and DILI-negative labels were obtained from curated resources used solely for evaluation; these labels are appropriate for hidden reference checks but do not replace expert causality adjudication for individual cases. The available panel provenance supports source-axis collapse counts, including concordant negative provenance where both DILIrank and DILIst negative axes were available, but it does not provide a version-matched granular source file sufficient to estimate full inter-source agreement across every DILIrank, DILIst, and LiverTox category. The evidence packets were based on PubMed title and abstract records rather than a full-text review of every source. Thus, “no direct evidence” means no direct DILI decision-support evidence in the supplied packet, not the absence of any possible evidence in labels, full text, adverse-event reports, or other resources. The recall stress test identified five additional direct-positive drugs from title/abstract records, confirming that the packet was not a complete literature-recall system; within this restricted channel, supplied-packet recall and precision are reported explicitly in the supplement. This limitation is relevant to deployment: an evidence-gated interface is only as safe as the retrieval layer feeding it. Future implementations should therefore extend retrieval auditing beyond title and abstract records to full text, product labels, and prospective retrieval-recall monitoring before clinical use. The three repeats mainly assessed response stability rather than creating independent observations: in closed-book analyses, the proportion of unanimous triplets was 89.5%–94.7% in the no-direct-evidence slice, 80.0%–100.0% in the positive/no-direct-evidence slice, and 84.0%–96.0% in the recent or low-recognition slice. We therefore averaged repeats within drug before inference and interpreted the effective unit as the drug. Model identities, provider interfaces, and default decoding behavior can change over time. We therefore report model provenance, decoding settings, and response files, but the results should be interpreted as a snapshot of the evaluated systems rather than a permanent ranking. Differences in provider-supported decoding controls also mean that comparisons should be read primarily as behavior under a fixed task protocol, not as a pure architecture comparison. The evidence-gated arm was evaluated as a structured protocol rather than a deployed clinical tool; real-world use would require human oversight, full-text retrieval, label review, adverse-event terminology normalization, and governance for model updates. We did not run a clinician-from-memory baseline on the identical 47-drug task; such a study would require a separate human-subject comparator design and is an important future benchmark for judging whether closed-book LLM behavior differs from unaided clinician memory.

These boundaries define the intended operating setting. In a controlled DILI risk-assessment task, closed-book LLMs frequently answered beyond the supplied evidence, including low-risk reassurance for hidden DILI-positive drugs. A simple evidence-gated protocol converted the same task into an auditable test of abstention, citation containment, and adherence to the supplied evidence grades, while preserving citation-grounded responses when direct evidence was provided. For drug-safety and regulatory uses of LLMs, the practical standard should therefore not be whether a model can produce a plausible answer, but whether the system can enforce and document that the answer stays within an externally supplied evidence boundary available to the user.

## Data Availability

The datasets presented in this study can be found in online repositories. The names of the repository/repositories and accession number(s) can be found at: Zenodo: https://doi.org/10.5281/zenodo.20587229.
